# Time-dependent inhibition of PHD2

**DOI:** 10.1042/BSR20170275

**Published:** 2017-06-30

**Authors:** Isabelle Tcholakov, Charles E. Grimshaw, Lihong Shi, Andre Kiryanov, Sean T. Murphy, Christopher J. Larson, Artur Plonowski, Jacques Ermolieff

**Affiliations:** 1 In Vitro Pharmacology, Immunology, Takeda California, Inc., 10410 Science Center Drive, San Diego, CA 92121, U.S.A.; 2 Enzymology and Biophysical Chemistry, Takeda California, Inc., 10410 Science Center Drive, San Diego, CA 92121, U.S.A.; 3 Medicinal Chemistry, Takeda California, Inc., 10410 Science Center Drive, San Diego, CA 92121, U.S.A.; 4 External Innovation, Takeda California, Inc., 10410 Science Center Drive, San Diego, CA 92121, U.S.A.; 5 Metabolic Disease, Takeda California, Inc., 10410 Science Center Drive, San Diego, CA 92121, U.S.A.; 6 In Vitro Pharmacology, Gastrointestinal and Enterology Discovery Unit, Takeda California, Inc., 10410 Science Center Drive, San Diego, CA 92121, U.S.A.

**Keywords:** hypoxia, hypoxia inducible factor, liquid chromatography-tandem mass spectrometry, prolyl hydroxylase 2, residence time, time-dependent inhibition

## Abstract

Prolyl hydroxylases (PHDs) down-regulate the level of hypoxia-inducible factors (HIFs) by hydroxylating key proline residues that trigger the degradation of the protein and affect the cell and its ability to respond to hypoxic stress. Several small molecule PHD inhibitors are now in various preclinical and clinical stages for the treatment of anemia. The present study provides a detail kinetic analysis for some of these inhibitors. The data generated in the present study suggest that these compounds are reversible and compete directly with the co-substrate, 2-oxoglutarate (2-OG) for binding at the enzyme active site. Most of these compounds are pan PHD inhibitors and exhibit a time-dependent inhibition (TDI) mechanism due to an extremely slow dissociation rate constant, *k*_off_, and a long residence time.

## Introduction

Cancer, stroke, and a variety of other diseases are often associated with hypoxic injuries that, ultimately, lead to tissue damage. To protect organs from this type of injury, the human body reacts by activating a number of cellular pathways under the regulation of hypoxia-inducible transcription factors (HIFs) and prolyl hydroxylase (PHD) enzymes. In response to hypoxia, HIF-1α regulates the transcription of a myriad of survival genes that will stimulate angiogenesis, cell proliferation, cell survival, and a variety of other pathways [1–3]. Conversely, PHD enzymes function as oxygen sensors that are responsible for the hydroxylation and degradation of HIF-1α. Upon hydroxylation, HIF-1α is ubiquitylated by the von Hippel–Lindau (VHL) factor that initiates its proteolytic degradation by the proteasome. Three different mammalian PHDs have been identified: PHD1, PHD2, and PHD3, along with a fourth enzyme, the factor-inhibiting hypoxia-inducible factor 1 (FIH1), also known as asparaginyl hydroxylase [2,3]. With the exception of FIH1, the rest of these enzymes display 45–65% sequence similarity and have some overlapping functions. All four enzymes require for their catalytic activity 2-oxoglutarate (2-OG) and oxygen as co-substrates, and ascorbic acid and iron (II) as cofactors. In addition, the expression location of PHD isoforms varies in different tissues and cell types. PHD2 and PHD3 mRNA are ubiquitously expressed with a higher level in adipose, heart, and placenta tissues, while PHD1 is more abundant in testis [4]. At the cellular level, Metzen et al. [5] showed that PHD1 is localized to the nucleus, while PHD2 and FIH1 are present in the cytoplasm, and PHD3 is equally distributed across both cellular compartments. More importantly, expression of the mRNA for PHD2 and PHD3 is induced under hypoxic conditions, while for PHD1 and FIH1, their mRNA expression level is independent of the oxygen level. At the protein level, FIH1 hydroxylates Asn^803^ and Asn^851^ residues located within the C-terminal domain of HIF-1α and HIF-2α respectively, whereas PHD1, PHD2, and PHD3 hydroxylate two proline residues (Pro^564^ and Pro^402^) located within the C-terminal half of HIF-1α [2]. Ultimately, the mechanism of hydroxylation of these residues regulates the level of HIF proteins in the cell [6]. Given such critical role of PHDs in regulating HIF levels and transcriptional activity, several small-molecule PHD inhibitors have been developed for the treatment of anemia and the prevention of hypoxic tissue injuries. These inhibitors comprise molecules of various chemotypes and differ in biochemical potencies related to diverse modes of kinetic interaction with the PHD enzymes.

The objective of the present study is to provide a comprehensive kinetic analysis of well-known PHD inhibitors, and by doing so we hope to provide some information about the key kinetic parameters associated with these compounds that drive their cell and *in vivo* potency. In addition, the present study should help to assess the kinetic profile of these inhibitors and provide additional information about their desired biochemical properties.

## Materials

**Figure 1:**
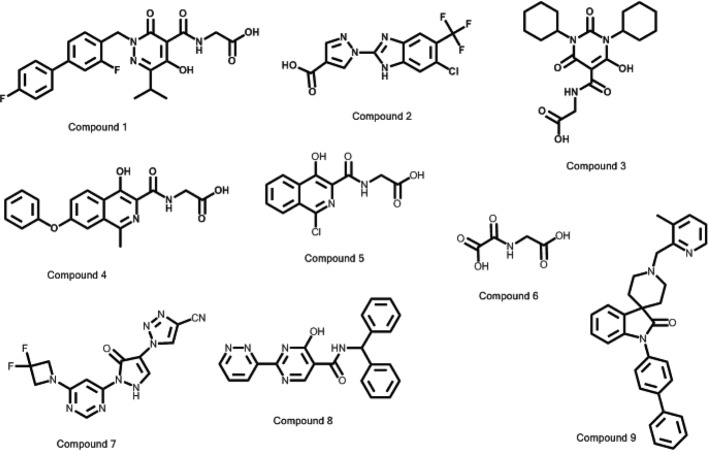
2D structures of PHD2 inhibitors analyzed in the present study The structure of compound **10** is not disclosed for proprietary reasons. General information about the chemical series developed by Takeda is provided in patent WO2014160810.

Active PHD1 (residues 163–403; 26.5 kDa, no tag), PHD2 (residues 180–417; 27.5 kDa; C-terminal His6-tagged), PHD3 (full length, 27.3 kDa, no tag), and FIH1 (full length, 40.4 kDa, no tag) were human recombinant proteins, expressed in *Escherichia coli* and purified in house at Takeda California (San Diego, U.S.A.). The substrate used for our PHD2 kinetic study was a 17-mer peptide mimicking the sequence of HIF-1a surrounding the Pro^564^ residue hydroxylated by the PHD enzymes (Biotin-DLEMLAPYI**P**MDDDFQL). The substrate used for FIH1 was a 34-mer peptide mimicking the sequence of HIF-1α surrounding residue Asn^803^ (DESGLPQLTSYDCEV**N**APIQGSRNLLQGEELLRAL). Both peptides were synthesized by CPC Scientific Inc. (Sunnyvale, CA, U.S.A.). All the compounds listed in Figure 1 were synthesized and purified in-house at Takeda California. Hepes and ascorbic acid were purchased from Fisher Scientific (Pittsburgh, PA, U.S.A.). Potassium chloride (KCl) and tris(2-carboxyethyl)phosphine hydrochloride salt (TCEP) were from Teknova (Hollister, CA, U.S.A.) and Thermo Fisher Scientific (Carlsbad, CA, U.S.A.) respectively. All other reagents were purchased from Sigma–Aldrich (St Louis, MO, U.S.A.). Unless stated otherwise, the kinetic studies listed in the experimental section were conducted at room temperature (∼22°C) using an assay buffer containing 50 mM Hepes, 50 mM KCl, 0.5 mM TCEP, 0.1 mg/ml BSA, and 2 μM FeCl_2_ at pH 7.3, except for the FIH1 assay buffer that contained 5 mM TCEP and 1 mM triglycine.

## Methods

### *K*_m_ determination for 2-oxoglutarate in the presence of PHD and FIH1 enzymes

For PHD and FIH1 enzymes, the Michaelis–Menten constant, *K*_m_, for 2-OG was determined by adding increasing amounts of 2-OG to a mixture containing a fixed amount of peptide substrate (1 μM) and ascorbic acid (1 mM) in assay buffer (see ‘Materials’ section). After 5–10 min incubation, the reaction was initiated by adding enzyme (50, 5, 40, and 15 nM for PHD1, PHD2, PHD3, and FIH1 respectively). The enzymatic reaction was set up in a 96-well plate and an aliquot was collected and analyzed at regular time intervals using a LEAP autosampler (CTC HTC PAL Leap Technologies Analytics Auto Sampler with Stack Cooler, Trajan Scientific and Medical, Victoria, Australia) coupled to an LC/MS/MS system (Agilent 1200 HPLC with AB Sciex API3000 triple quadrupole mass spectrometer). The enzyme activity was determined by measuring the rate of formation of the hydroxylated HIF peptide product over time. The hydroxylated product was separated via an Aeris WIDEPORE 3.6u, XB-C-18 column (Phenomenex, Torrance, CA, U.S.A.) using a solution of 0.5% formic acid in water (mobile phase A) and 0.5% formic acid in acetonitrile (mobile phase B), with the following gradient: 0.4 min, 2% B; 0.41 min, step gradient up to 30% B; 0.41–1.42 min; linear gradient from 30% to 75% B; 1.42–1.6 min; linear gradient from 75% to 98% B; 1.6–1.89 min, 98% B; 1.95–2.1 min, back to 2% B. The flow rate was 1 ml/min, except between 1.6 and 1.89 min when the flow rate was lowered to 0.6 ml/min. The product of the PHD1, 2, and 3 reaction was detected in a multiple reaction monitoring (MRM) experiment with precursor molecular ion (Q1) of 1283.10 Da and fragment ion (Q3) of 1115.90 Da. Substrate disappearance was also monitored by MRM mode as well with precursor ion (Q1) of 1277.70 Da and fragment ion (Q3) of 1111.20 Da. FIH1 substrate disappearance and product formation were detected by monitoring MRM ion transitions Q1/Q3 of 1127.400/1355.50 and 1135.60/1370.60 Da respectively. The API3000 instrument was set up in positive ion mode and data were recorded and analyzed using SCIEX Analyst 1.5 and SCIEX MultiQuant 3.0.1 software packages.

### IC_50_ determination and time dependency

**Figure 2:**
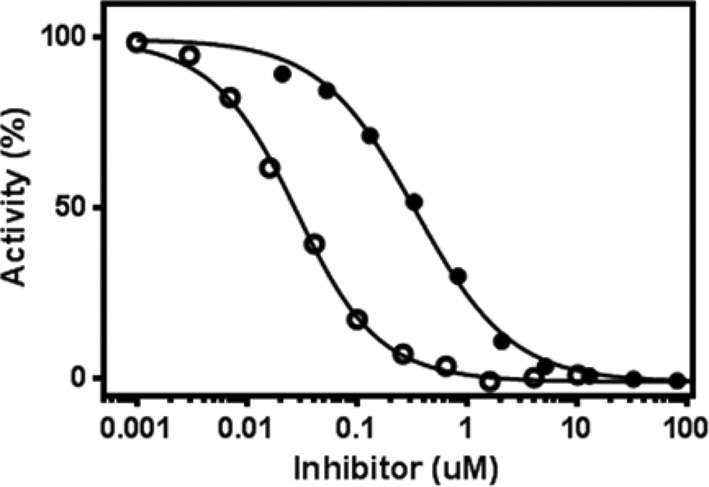
Time-dependent inhibition The IC_50_ values of compound **4** against PHD2 were determined without (●) and with preincubating (○) the enzyme and the inhibitor together in the assay buffer for 1 h prior to the addition of the substrate.

The potency of the compounds used in the present study (see Figure 1) was determined by measuring the rate of product formation in the presence of increasing concentration of inhibitor mixed with a fixed concentration of enzyme (25, 5, 10, and 15 nM for PHD1, 2, 3, and FIH1 respectively). The reaction was initiated by adding a fixed concentration of 2-OG (2 and 0.7 μM for PHDs and FIH1 respectively), ascorbic acid (1 mM), and peptide (1 μM) in assay buffer. The reaction was stopped after 1 h by adding an excess of a potent control inhibitor for each enzyme. The formation of product and the disappearance of substrate were detected using an LC/MS/MS instrument as previously described (see ‘Methods’ section—*K*_m_ determination). Experimental data were fitted using eqn (1) (Figure 2).

1 $$\frac{V_{i}}{V_{o}}=\frac{100}{1+{\left(I/{\mathrm{IC}}_{50}\right)}^{n}}$$

Where *V*_i_ and *V*_o_ are the rates of substrate hydroxylation in the presence or absence of inhibitor respectively; *n* is the Hill coefficient; I is the free inhibitor concentration; and IC_50_ is the measure of potency equivalent to the inhibitor concentration that leads to a 50% inhibition of enzyme activity.

In order to assess the time-dependent inhibition profile for each of these inhibitors, IC_50_ values were determined with and without preincubating enzyme and inhibitor together in the assay buffer for 1 h prior to the addition of 2-OGsubstrate (Figure 2). Both sets of IC_50_ values are reported in Table 1.

**Table 1:** Kinetic and selectivity data for a series of PHD small molecule inhibitors

Compound ID	PHD2				PHD1	PHD3	FIH1
	IC_50_ (μM)—no incubation	IC_50_ (μM)—60 min incubation (*n*-fold change)	*k*_off_ (h^-1^)	τ (h)	IC_50_ (μM)—60 min incubation	IC_50_ (μM)—60 min incubation	IC_50_ (μM)—60 min incubation
**1**	0.284	0.019 (14.9)	0.240	4.3	0.003	0.013	1.445
**2**	0.440	0.039 (11.3)	0.010	105	0.011	0.025	5.495
**3**	0.510	0.073 (7)	0.103	9.7	0.015	0.008	3.631
**4**	0.308	0.030 (10.3)	0.120	8.3	0.002	0.005	>10
**5**	0.560	0.324 (1.7)	fast	<1	0.016	0.021	>10
**6**	0.949	1.549 (0.6)	fast	<1	0.851	0.186	0.191
**7**	0.034	0.009 (3.8)	0.49	2.0	0.020	0.012	0.955
**8**	0.036	0.029 (1.2)	fast	<1	0.020	0.025	9.772
**9**	0.204	0.158 (1.3)	fast	<1	1.621	2.042	>10
**10 (Takeda)**	0.066	0.005 (16.5)	0.002	500	0.009	0.009	5.128

### Mechanism of inhibition and *K*_i_ determination for a selected set of compounds in the presence of PHD2

**Figure 3:**
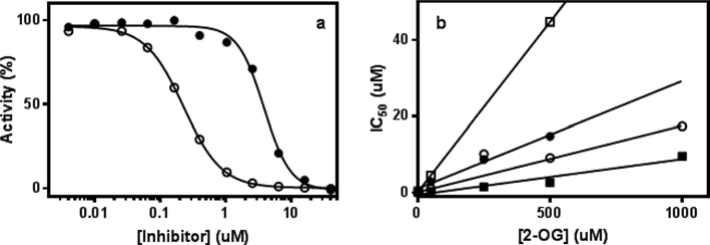
Mechanism of inhibition and K_i_ determination (**a**) Inhibition of PHD2 by compound **2** was evaluated by measuring IC_50_ values in the presence of 2 μM (●) and 50 μM (○) of 2-OG. (**b**) The mechanism of inhibition of compounds **2** (**●**), **3** (**○**), **4** (**□**), and **7** (▪) against PHD2 was determined by measuring IC_50_ values in the presence of increasing 2-OG concentrations (from 0.002 to 1 mM 2-OG, at least three concentrations of 2-OG were used in the present study) without preincubation.

The mechanism of inhibition of compounds **2**, **3**, **4**, and **7** against PHD2 was determined by measuring IC_50_ values without preincubation in the presence of increasing 2-OG concentrations (from 0.002 to 1 mM 2-OG, at least three concentrations of 2-OG were used for the study—see Figure 3). The experimental data were fitted using eqn (2) for a competitive inhibitor.

2 $${\mathrm{IC}}_{50}=K_{i}\cdot \left(1+\frac{S}{K_{m}}\right)$$

where *S* is the total concentration of 2-OG and *K*_m_ is the Michaelis–Menten constant for 2-OG.

### Direct measurement of the dissociation rate constant, *k*_off_, for PHD2

**Figure 4:**
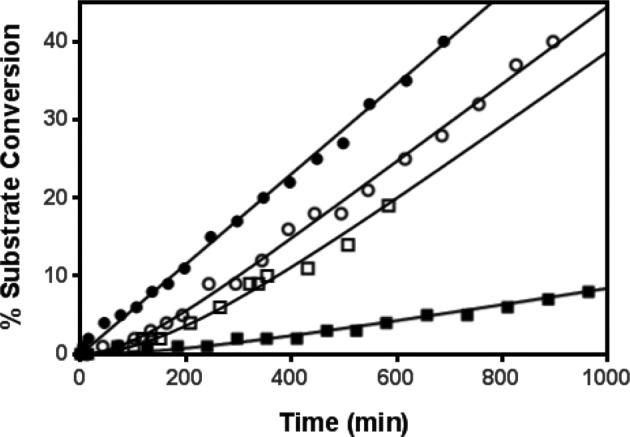
Rapid dilution experiment for the determination of *k*_off_ constants Inhibitors were preincubated with PHD2 to form a complex E–I. An aliquot of this mixture was the rapidly diluted a large volume of buffer containing an excess of 2-OG substrate. Off-rate constants for compound **1** (○), **2** (▪), and **3** (□) were determined by measuring the activity with time of the free enzyme releases from the complex E–I. For comparison purposes, experimental data for the enzyme alone (●) have been reported in the same figure.

The dissociation rate constant for each inhibitor against PHD2 enzyme was determined using a rapid dilution experiment. The assay was conducted by mixing equimolar amounts of enzyme (E; 1 μM) and inhibitor (I; 1 μM). After 2-h preincubation, aliquots of the mixture were transferred to a separate 96-well plate and diluted 500-fold into assay buffer in the presence of a fixed concentration of peptide substrate (1 μM), an excess of 2-OG (500 uM), and ascorbic acid (1 mM). The release of the inhibitor from the preformed E–I complex was monitored by recording reaction product formation over time as shown in Figure 4. Dissociation time-course data were fitted to determine *k*_off_ and residence time, τ, using eqns (3) and (4) respectively.

3 $$P=V_{s}\cdot t-\frac{V_{s}}{k_{\mathrm{off}}}\cdot (1-e^{-k_{\mathrm{off}}\cdot t})$$

4 $$\tau =\frac{1}{k_{off}}$$

where *P* is the concentration of the hydroxylated peptide product generated at time *t*, and *V*_s_ is the rate of hydroxylated product formation by uninhibited PHD2 at steady state. The *k*_off_ and residence time, τ, values determined are listed in Table 1.

## Results

### *K*_m_ for 2-OG, reversibility, and mechanism of inhibition

For comparison purposes, the 2-OG *K*_m_ value was determined for each of the PHD and FIH1 enzymes under the same experimental conditions (see ‘Methods’ section). *K*_m_ values determined under these conditions are consistent with the ones previously published [6–10] and are not significantly different across all the PHD isotypes used in the present study: 0.31, 0.98, 0.66, and 0.56 μM for PHD1, 2, 3, and FIH1 respectively. In addition, we have shown that these compounds are reversible inhibitors for PHD2. The reversibility study was conducted by diluting the E–I complex in the presence of a large volume of buffer and an excess of substrate. Under these conditions, the inhibitor is slowly released from the E–I complex and the recovered enzyme activity is monitored by measuring the formation of product (i.e. hydroxylated peptide) with time as illustrated in Figure 4. These experimental data allow us to determine the *k*_off_ constant for the mechanism of interaction of these inhibitors with PHD2. Results reported in Table 1 show that values of the dissociation rates vary greatly for all the compounds tested. The reversible inhibition mechanism of these compounds was only evaluated in the presence of PHD2; nevertheless, we hypothesize that most of these inhibitors behave similarly with the other PHD isotypes, including FIH1. The mechanism of inhibition of these compounds was further investigated in the presence of several concentrations of 2-OG substrate. As illustrated in Figure 3(b), IC_50_ values for compounds **2**, **3**, **4**, and **7** increase linearly with increasing 2-OG substrate concentration suggesting that they compete with 2-OG substrate for binding at the enzyme active site. These results are in agreement with published studies showing that compound **2** is a reversible inhibitor and competes with 2-OG [8,9]. From the data illustrated in Figure 3(b), we have also determined an inhibition constant *K*_i_ for these four inhibitors using eqn (2). The calculated *K*_i_ values are 0.027, 0.017, 0.009, and 0.087 μM for compounds **2**, **3**, **4**, and **7** respectively.

### Time-dependent inhibition, residence time, and off-rate

All the compounds listed in the present study were also evaluated for their time-dependent inhibition mechanism against PHD2. Briefly, the study was set up to measure the potency of these compounds with and without preincubation with the enzyme. As illustrated in Figure 2 and Table 1, some of these compounds (i.e. compounds **1**–**4**, **7**, and **10**) showed a significant increase in potency after 60 min preincubation (i.e. IC_50_ decreased by at least 5-fold), which suggests that the formation of E–I complex is slow to reach equilibrium. Time-dependent inhibitor binds to, or dissociate slowly from their target and in order to further investigate this mechanism, we have determined the dissociation rate constant, *k*_off_, and the residence time, τ, for each of these compounds in the presence of PHD2. As reported in Table 1, the residence time varies greatly across this set of inhibitors from less than 1 h to more than several days. One of the compounds with the longest residence time is inhibitor **10** (structure not disclosed), a proprietary Takeda compound. For the time being, we can only speculate about the causes of this time-dependent inhibition as it remains unclear which are the key pharmacophores, chemical motifs, and/or critical residues in the enzyme-binding pocket that are responsible for the observed time-dependent inhibition of PHD2.

### Selectivity screening

The compounds in the present study were also screened against PHD1, PHD3, and FIH1 using a 60-min preincubation. As reported in Table 1, these compounds show various degrees of potency toward PHD1 and/or PHD3 despite a highly conserved catalytic domain with PHD2. Conversely, none of the compounds tested significantly inhibit FIH1.

## Discussion

PHD enzymes and HIF transcription factors are essential to maintain cell viability under hypoxic conditions. The modulation of the activity of these enzymes could be beneficial for the treatment of a variety of diseases, therefore, a large number of PHD2 inhibitors have been developed and are now in late stage clinical trials for the treatment of anemia and chronic kidney diseases [8–13]. Several companies have reported PHD inhibitors in clinical development for the treatment of ischemia and anemia associated with chronic renal disease. The goal of the present study is to provide a detail kinetic profile for some of these well-known PHD2 inhibitors and to investigate which key kinetic microscopic parameters predict their efficacy beyond the simple measure of potency. Our initial results showed that these compounds compete with 2-OG at the enzyme active site and thus their apparent potency is adversely affected by high concentrations of this co-substrate in the media (see Figure 3b) and probably also in cells [14]. Most interestingly, a few of these compounds are tight binding inhibitors and display a slow onset of inhibition. As illustrated in Table 1, the IC_50_ for compounds **1**–**4**, **7**, and **10** decreased significantly (by at least 5-fold) when preincubated for 60 min in the presence of PHD2. We further investigated this slow binding mechanism by measuring the dissociation rate constant for all the compounds used in the present study. Interestingly, each of the inhibitors that displayed a time-dependent inhibition mechanism is associated with a slow dissociation rate constant (or the long residence time—see Table 1), suggesting that these compounds are slow to reach equilibrium with their binding partner. The underlying causes for these slow dissociation rates remain to be fully understood, however, several interesting hypotheses have emerged from the literature. For example, Pan et al. [15] suggested that the flexibility of the ligand and/or receptor can have an impact on these microkinetic constants. Other studies [16,17] have shown that an enzyme in solution can exist in several conformation states and can bind to a ligand (substrate and inhibitor) according to an induced-fit and/or a conformational recognition mechanism, both associated with different kinetic constants. During the conformational process, the enzyme has already adopted a competent conformation and will not experience significant structural changes to initiate a productive binding as opposed to the induced-fit mechanism. Interestingly, the plasticity of the PHD2 active site has been well documented by Chowdhury et al. [18,19], whose team showed that the binding of a peptide substrate stabilizes the β2/β3 loop region and close the access to the active site. According to these authors, these changes of conformation are critical to stabilize the PHD2–Fe(II)–2OG complex and are necessary to achieve full enzyme activity. Despite the lack of supporting structural evidence, we propose that most of the PHD2 inhibitors used in this study shift the equilibrium of the free enzyme population to favor an induced-fit mechanism leading to significant conformation changes in the β2/β3 loop region to lock these inhibitors inside the active site and slow the rate of their release from the enzyme. This entrapment mechanism can also prevent access of the solvent to the active site strengthening potential hydrophobic, hydrogen, and Van der Waals interactions that might exist between the ligand and the receptor [15].

Several studies have shown that compounds with a long residence time display greater cell or *in vivo* potency. In a recent review, Copeland [20] summarized some of the recent findings in this field. Interestingly, the same review also addresses how mutations within the target binding sites, and/or slight changes in the drug chemical structure can affect the rate of dissociation, *k*_off_, for these molecules. Similarly, in the present study compounds **7** and **10**, which originate from two different chemotypes, revealed a striking 250-fold difference in residence time despite showing similar IC_50_ values. As previously suggested, compounds with the longest residence time could provide the best therapeutic effect; however, the impact of this parameter upon the *in vitro* and *in vivo* potencies for a drug could be limited by several factors such as the half-life of the protein target and the pharmacokinetic half-life of the drug itself. For the PHD enzymes, several publications have determined a half-life between 2 and 48 h in cells under various stimuli [21,22], suggesting that compounds with a residence time longer than 3 days may not show any additional *in vivo* pharmacodynamic benefits. In addition, a longer residence time could also have a negative impact on the safety profile of these drugs due to unwanted on-target effects.

Promising studies have also shown that these compounds could present some benefits for other diseases such as inflammatory bowel disease and Crohn’s disease where the inflammatory process is frequently associated with hypoxia caused by a lack of oxygen supply and an increase in metabolic demands in the intestinal tissue. Hence, the benefit of inhibiting PHD2 by small molecules could extend far beyond the treatment of anemia [23,24]. The challenge is to balance the benefits and the hindrances provided by these compounds. More specifically, we need to understand which chemical and biophysical parameters need to be optimized to achieve the optimal efficacy. To that extent, we have provided a detail kinetic analysis for a few well-known PHD2 inhibitors. Our data show that some of these inhibitors are slow to reach binding equilibrium with their target and also show extremely slow off-rates and long residence times. Finally, the present study can provide insight about key kinetic parameters that affect the *in vivo* potency of these compounds. Rational utilization of binding kinetics in drug optimization can yield the next generation of improved PHD inhibitors.

## Abbreviations

2-OG: 2-oxoglutarate
BSA: bovine serum albumin
FIH1: factor-inhibiting hypoxia-inducible factor 1
HIF-1α: hypoxia-inducible factor-1α
IC_50_: concentration of inhibitor giving 50% inhibition of enzyme activity
LC/MS: liquid chromatography/mass spectrometry
MRM: multiple reaction monitoring
PHD: prolyl hydroxylase
TCEP: tris(2-carboxyethyl)phosphine
TDI: time-dependent inhibition
VHL: von Hippel–Lindau
